# Carbon and Heteroatom Activation via *N*‐Heterocyclic Carbene‐Bound Dearomative Intermediates for Stereoselective Annulation Reactions

**DOI:** 10.1002/chem.202502375

**Published:** 2025-10-24

**Authors:** Carmela De Risi, Graziano Di Carmine, Daniele Ragno, Lorenzo Poletti, Alessandro Massi

**Affiliations:** ^1^ Dipartimento di Scienze Chimiche, Farmaceutiche ed Agrarie Università di Ferrara Via L. Borsari, 46 Ferrara 44121 Italy; ^2^ Dipartimento di Scienze dell'Ambiente e della Prevenzione Università di Ferrara Via L. Borsari, 46 Ferrara 44121 Italy

**Keywords:** annulation, C‐H activation, *N*‐Heterocyclic carbenes, organocatalysis, stereoselective synthesis

## Abstract

*N*‐Heterocyclic carbene (NHC) organocatalysis has long been recognized as a powerful and versatile method for the implementation of (asymmetric) transformations enabling the formation of C─C and C–heteroatom bonds. A number of NHC‐bound active ionic species have been classically established for activation of carbon atoms as either nucleophiles or electrophiles at diverse positions, such as (*aza*)Breslow intermediates, homoenolate equivalents, azolium (di)enolates, simple acyl (imidoyl) azoliums, and α,β‐unsaturated/alkynyl acyl azoliums. Within this realm, the past decade has witnessed a blossoming interest in the deployment of NHC‐tethered dearomative intermediates for the activation of both carbon atoms and heteroatoms in aromatic compounds. Accordingly, functionalization of benzylic carbon atoms was made possible through catalyst‐bound *o*‐quinodimethanes (*o*‐QDMs), while remote activation of oxygen and nitrogen atoms was effectively achieved through NHC‐bound *o*‐quinone methides (*o*‐QMs) and *aza*‐analogs (*aza*‐*o*‐QMs), along with *aza*‐fulvene type intermediates and triaza‐diene species. Aim of this review is to provide an overview of the most relevant literature on the development of NHC‐bound dearomative intermediates for carbon and heteroatom activation since 2013, with emphasis on their applications for stereoselective annulation reactions.

## Graphical abstract

Use of *N*‐heterocyclic carbenes (NHCs) to activate carbon and heteroatoms in aromatic compounds is reviewed based on key dearomative NHC‐bound intermediates, i.e., *ortho*‐quinodimethanes (*o*‐QDMs), *ortho*‐quinone methides (*o*‐QMs) and *aza*‐analogs (*aza*‐*o*‐QMs), *aza*‐fulvene and triaza‐diene intermediates. Their formation and reactivity in stereoselective annulation reactions for the synthesis of enantioenriched (hetero)cyclic molecules are discussed.

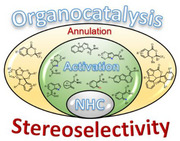

## 1 Introduction

**Figure 1:**
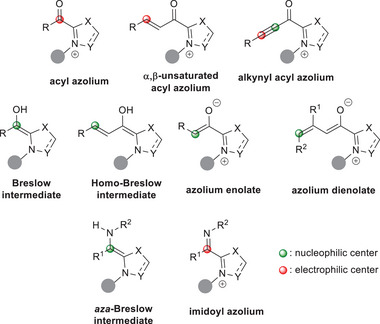
Typical NHC‐bound ionic intermediates for carbon atom activation.

It is now established that catalysis by (chiral) *N*‐heterocyclic carbenes (NHCs) is a potent tool in the hands of synthetic organic chemists for the accomplishment of very diverse (stereoselective) transformations through distinctive modes of substrate activation.^[^ ^1^, ^2^, ^3^, ^4^, ^5^, ^6^, ^7^, ^8^, ^9^, ^10^, ^11^, ^12^, ^13^, ^14^, ^15^, ^16^ ^]^ NHCs make possible to activate carbon atoms as both electrophiles and nucleophiles by leveraging typical NHC‐bound ionic intermediates, inter alia, acyl^[^ ^17^ ^]^/imidoyl^[^ ^18^, ^19^ ^]^ azolium species, α,β‐unsaturated^[^ ^17^, ^20^, ^21^, ^22^, ^23^ ^]^/alkynyl^[^ ^24^, ^25^, ^26^ ^]^ acyl azoliums, acyl anion equivalents (Breslow intermediates)^[^ ^27^, ^28^, ^29^, ^30^, ^31^, ^32^, ^33^, ^34^ ^]^ and their *aza*‐analogs (*aza*‐Breslow intermediates),^[^ ^35^, ^36^, ^37^, ^38^, ^39^ ^]^ homoenolate equivalents (Homo‐Breslow intermediates),^[^ ^40^, ^41^, ^42^, ^43^ ^]^ as well as azolium enolates^[^ ^44^, ^45^, ^46^, ^47^, ^48^ ^]^ and azolium dienolates (Figure 1).^[^ ^46^, ^49^, ^50^ ^]^

**Scheme 1:**
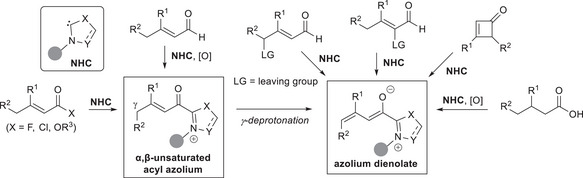
Most common routes for the formation of NHC‐bound dienolates.

First reported by Ye and coworkers in 2011,^[^ ^51^ ^]^ azolium dienolates represent versatile 4C‐nucleophilic synthons for functionalization at either α‐ and remote γ‐positions, thus enabling to extend the classical azolium enolate chemistry in a vinylogous mode. As depicted in Scheme 1, azolium dienolates are typically built by γ‐deprotonation of α,β‐unsaturated acyl azolium intermediates, that can be reached from unsaturated carboxylic acid derivatives or by external oxidation of enal‐derived Breslow intermediates. Other than these approaches, some other modes of activation are also worthy of mention, including elimination reactions of enals bearing leaving groups at the α‐ or γ‐positions, activation of saturated carboxylic acids under oxidative conditions, and ring‐opening of cyclobutenones via selective C─C bond cleavage. Of note, each such protocol shows advantages and drawbacks that could be traced to the procedures used for forming other classical NHC‐bound intermediates.^[^ ^8^, ^46^ ^]^ Hitherto, the chemistry of chiral azolium dienolates has found mainly applications in enantioselective α‐halogenation or α‐alkylation and cycloadditions involving the remote γ‐carbons and the carbonyl moiety.^[^ ^49^ ^]^

**Scheme 2:**
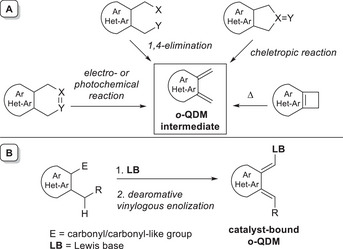
A) Classical methods for obtaining *o*‐QDM intermediate and B) formation of catalyst‐tethered *o*‐QDM by covalent organocatalysis.

Moreover, it must be said that the chemistry of azolium dienolates was mainly enacted on acyclic (nonaromatic) substrates, and has been significantly extended to aromatic molecules only in recent years. In this scenario, a possible combo of NHC and dearomatized *o*‐quinodimethane (*o*‐QDM) intermediate (Scheme 2) gained attention for the challenging functionalization of benzylic carbon atoms of (hetero)aryl carbonyl compounds.

First introduced by Cava and Napier in 1957,^[^ ^52^ ^]^ *o*‐QDM (*o*‐xylylene) species have since caught growing attention from the research community. From simple short‐lived intermediates, their use has evolved into highly activated diene synthons for the synthesis of (biologically relevant) benzannulated products via inter‐ or intramolecular [4 + 2] Diels‐Alder cycloaddition, as well as for the production of polymers and π‐extended materials.^[^ ^53^, ^54^, ^55^, ^56^, ^57^ ^]^

*o*‐QDM intermediates are classically formed by in situ dearomatization of a suitable (hetero)aromatic substrate via 1,4‐elimination, cheletropic reaction, and electro‐ or photochemical processes, or through ring opening of a cyclobutene‐fused aryl (Scheme 2A), but milder and more versatile methods have been also established to prevent the harsh reaction conditions often required for these transformations. In that respect, organocatalysis^[^ ^58^, ^59^ ^]^ offers the chance to access the *o*‐QDM species by dearomative vinylogous enolization of an aromatic carbonyl or carbonyl‐like compound bearing an appropriate substituent at *ortho* position: its benzylic C─H bond may take part in remote enolization and subsequent in situ functionalization with suited electrophiles, thereby preventing the strong tendency of *o*‐QDM to rearomatize.

It's important to point that heterocyclic *o*‐QDMs form much easier and are more stable than the carbocyclic analogs, due to the lower aromaticity of heterocycles in relation to benzene.^[^ ^60^ ^]^ In particular, benzofused heterocycles (e.g., indole, benzofuran, or benzothiophene derivatives) are more smoothly turned into the corresponding *o*‐QDMs, either by direct base‐catalyzed enolization (if very poorly aromatic) or through proper activation of the built‐in electrophilic moiety.

**Scheme 3:**
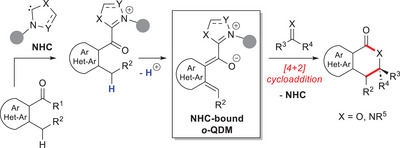
General route to NHC‐bound *o*‐QDM and its expected reactivity.

To date, organocatalytic generation of *o*‐QDMs has been mostly exploited by covalent activation modes (HOMO‐raising strategies), using chiral Lewis base (LB) promoters, especially amines and NHCs.^[^ ^61^, ^62^ ^]^ Reaction of the latter with properly functionalized (hetero)aromatic nuclei, followed by benzylic deprotonation, enables the formation of catalyst‐bound *o*‐QDM species (Scheme 2B). With particular reference to NHC organocatalysis, its use to generate *o*‐QDMs has spread starting in the second decade of the 2000s. Here, a pronucleophilic (hetero)aromatic carbonyl compound reacts with the active carbene species producing a transient acyl azolium intermediate which goes through dearomative vinylogous enolization to form an NHC‐bound *o*‐QDM diene, eventually reacted with activated C = O (or C = N) bonds in (stereoselective) formal [4 + 2] cycloaddition reactions (Scheme 3).

**Scheme 4:**
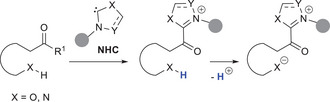
Remote activation of heteroatoms by NHCs.

In the domain of NHC‐catalyzed asymmetric reactions, the prevailing studies on nucleophilic activation of carbon atoms was flanked by a burgeoning interest in activation of heteroatoms over the last decade, albeit still less common.^[^ ^63^ ^]^ In that case, an NHC catalyst is covalently tied to (hetero)aromatic substrates that bear N─H and O─H moieties in apt position: this boosts their acidity, thus raising the “formal” nucleophilicity of nitrogen and oxygen atoms (Scheme 4).

**Figure 2:**
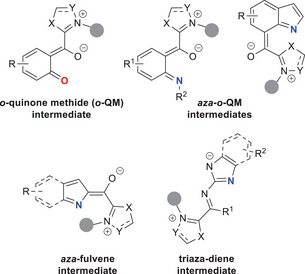
Typical NHC‐tethered intermediates for activation of heteroatoms.

More specifically, activation of nitrogen and oxygen atoms for enantioselective reactions was first reported through *o*‐quinone methides (*o*‐QMs)^[^ ^64^, ^65^ ^]^ and *aza*‐analogs (*aza*‐*o*‐QMs), that represent azolium dienolate analogs where heteroatoms replace carbon atoms at the γ‐position. Further studies demonstrated that activation of nitrogen atoms could be further realized via *aza*‐fulvene intermediates and triaza‐diene species (Figure 2). Similar to *o*‐QDMs, all these NHC‐bound intermediates were successfully tested in annulation reactions with electrophilic ketone (imine) substrates.

Since 2019, formation of NHC‐bound dearomative intermediates for carbon and heteroatom activation, and their possible application in annulation reactions have been addressed in different review articles. Chen and coworkers discussed asymmetric reactions promoted by *o*‐QDMs and *o*‐QMs,^[^ ^61^ ^]^ while Marcantonio and Curti presented stereoselective applications of heterocyclic *o*‐QDMs.^[^ ^62^ ^]^ Chi group surveyed NHC‐catalyzed activation of heteroatoms for enantioselective reactions,^[^ ^63^ ^]^ Zhong and Yang focused on NHC‐activated *o*‐aryldiquinoid and triaza‐diene intermediates as part of NHC‐catalyzed activation strategies,^[^ ^12^ ^]^ and the same was done by De Risi and colleagues in the context of oxidative *N*‐heterocyclic carbene catalysis.^[^ ^66^ ^]^

Other than that, some more works on the topic appeared in the literature, also investigating mechanisms, origin of selectivities, and catalyst(s) role. Even so, a review giving an overall look on activation of both carbon and heteroatoms through NHC‐based dearomative species for annulation reactions seems to lack, to the best of our knowledge. On that basis, we aim to give a new picture of this subject reviewing the literature produced from 2013 to date. It must be said that we made the choice to focus exclusively on stereoselective approaches, privileging the most common kinds of carbon and heteroatom activation via NHC‐tied dearomative species, while other rarer types of activation, including those on δ‐^[^ ^67^ ^]^ and ε‐position,^[^ ^68^ ^]^ are not discussed in this article.

## 2 Activation of Carbon Atoms and Heteroatoms Through Dearomative Intermediates Covalently Bound to the NHC Catalyst

### 2.1 Activation of Carbon Atoms via NHC‐bound *o*‐QDMs

**Scheme 5:**
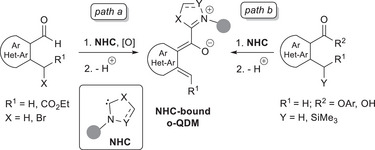
Main strategies for forming NHC‐bound *o*‐QDMs.

Usually, two routes mainly exist to form *o*‐QDMs from benzaldehydes and chiral NHCs, harnessing either *ortho*‐C─H or *ortho*‐C─Br bond activation (Scheme 5, *path a*), while an alternative *ortho*‐C─H bond activation may be carried out on analog aryl substrates appended with an ester moiety (Scheme 5, *path b*). Concerning this last route, generation of NHC‐bound *o*‐QDM was also possible via remote activation of C─H and C─Si bonds using 2‐methyl‐3,5‐dinitrobenzoic acid^[^ ^69^ ^]^ and 2‐[(trimethylsilyl)methyl]benzoate,^[^ ^70^ ^]^ respectively, nonetheless, these protocols have not been further expanded for stereoselective reactions.

**Scheme 6:**
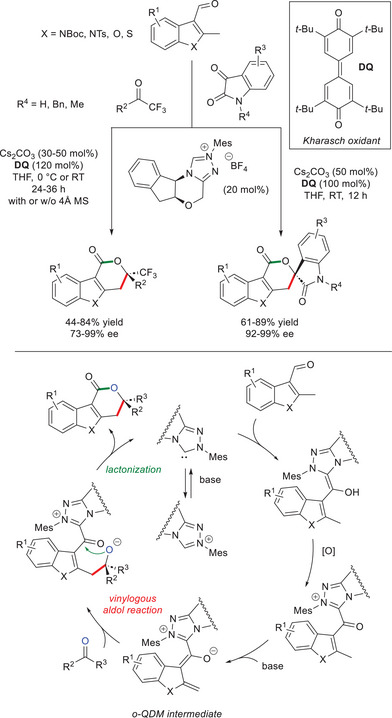
Enantioselective synthesis of polycyclic lactones via *o*‐QDMs derived from 2‐methylindole‐3‐carboxaldehydes.

In 2013, Chi and colleagues reported for the first time the use of NHC organocatalysis for the activation (functionalization) of benzylic C(sp^3^)─H bonds of heteroaryl aldehydes.^[^ ^71^ ^]^ Actually, α‐branched *N*‐protected indole 3‐carboxaldehydes, as well as benzofuran and benzothiophene analogs, were reacted with (hetero)aryl/alkyl trifluoromethyl ketones in the presence of chiral aminoindanol‐derived precatalyst (20 mol%) and cesium carbonate (Cs_2_CO_3_, 30–50 mol%) under oxidative conditions (Kharasch reagent, 3,3′5,5′‐tetra‐*tert*‐butyldiphenoquinone, **DQ**, 120 mol%),^[^ ^66^, ^72^ ^]^ with 4 Å molecular sieves (MS) added in the case of alkyl ketones (Scheme 6). Thus, polycyclic lactones with a quaternary stereocenter were obtained in low to good yields (44–84%) and good to excellent enantioselectivities (73–99% ee). Slightly modified conditions have also proved effective to achieve spirocyclic lactones (61–89% yield, 92–99% ee) using both unprotected and *N*‐protected isatins as the ketone components. Mechanistically, it is likely that the in situ generated heterocyclic *o*‐QDM takes part in a vinylogous aldol reaction followed by intramolecular lactonization with concurrent NHC displacement.

It is worth noting that the heterocyclic nuclei showed a unique reactivity against phenyl groups. As a matter of fact, 2‐methylbenzaldehyde gave no trace of the expected lactone, and its partial oxidation to the corresponding benzoic acid was exclusively observed under diverse conditions. This result can be accounted for by the very low C─H acidity at the remote γ‐position in the anticipated acyl azolium intermediate derived from the carbocyclic aromatic aldehyde, due to greater resonance stabilization of the benzene ring.

**Scheme 7:**
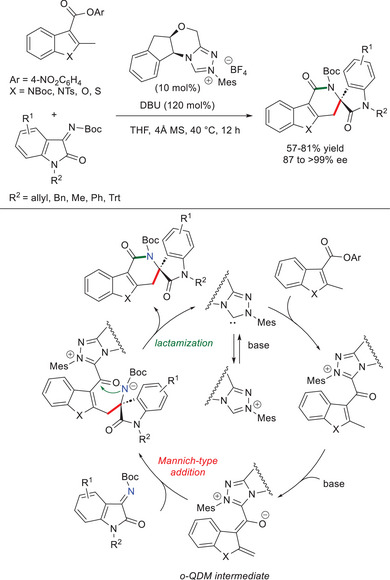
Synthesis of chiral heteroarene‐fused δ‐lactams via *o*‐QDMs derived from 2‐methyl‐heteroarene‐3‐carboxylic esters.

In 2016, Xu and coworkers described *o*‐QDM analogs derived from 2‐methyl‐heteroarene‐3‐carboxylic esters, and their enantioselective reactions with isatin‐derived ketimines to afford enantiomerically enriched heteroarene‐fused δ‐lactams (up to > 99% ee) incorporating a quaternary stereocenter (Scheme 7).^[^ ^73^ ^]^ A catalytic cycle was postulated where *o*‐QDM intermediate is involved in a Mannich‐type addition/lactamization sequence, with final regeneration of the NHC catalyst. An optimized protocol was identified involving the opposite enantiomer of the catalyst used by Chi and coworkers (10 mol%)^[^ ^71^ ^]^ and 1,8‐diazabicyclo[5.4.0]undec‐7‐ene (DBU, 120 mol%): a wide range of substituents on both lactam ring and phenyl nucleus of isatins were tolerated, with the highest enantioselectivities ensured by the *N*‐trityl (Trt) protecting group.

**Scheme 8:**
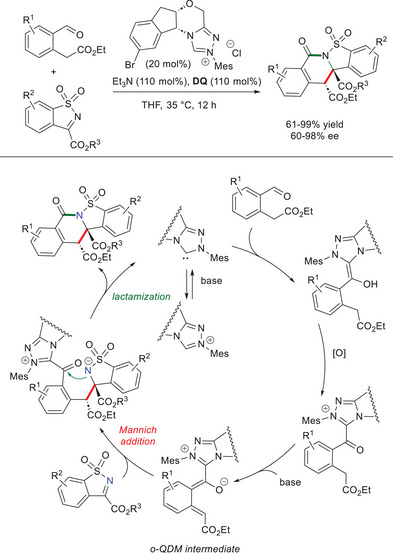
Synthesis of chiral dihydroisoquinolinones via *o*‐QDMs derived from formyl phenylacetic esters.

In the years since, efforts were pivoted toward effective strategies for producing the challenging carbocyclic *o*‐QDMs, starting from aryl carbonyls. Always riding on the acidity of acyl azolium intermediates at γ‐position, Zheng and coworkers took advantage of aryl aldehydes bearing an ester moiety at the benzylic carbon, so as to increase the acidity at its C─H bond.^[^ ^74^ ^]^ The ensuing *o*‐QDMs were engaged in enantioselective reactions with cyclic sulfonic imines to obtain chiral dihydroisoquinolinones (61–99% yield, 60–98% ee) with complete diastereoselectivity (Scheme 8). Optimal reaction conditions were found using a chiral NHC catalyst containing the (1*R*,2*S*)‐aminoindanol core (20 mol%), together with triethylamine (Et_3_N, 110 mol%) and **DQ** (110 mol%). The results were independent of the type and position of substituents on either the formyl phenylacetic ester or cyclic imine, with the exception of sterically hindered groups (i.e., OCH_3_ group on the aryl aldehyde at C6). Studies by electrospray ionization high‐resolution mass spectrometry (ESI‐HRMS) supported a stepwise process featuring a Mannich addition and an intramolecular amidation (*N*‐acylation), albeit a [4 + 2] concerted route was not totally ruled out. Of interest is that large‐scale (1.0 mmol) experiments have been successful without affecting enantioselectivity (71% yield, 96% ee), and various synthetic transformations (e.g., reduction, decarboxylation) of the obtained chiral dihydroisoquinolinones were possible.

**Scheme 9:**
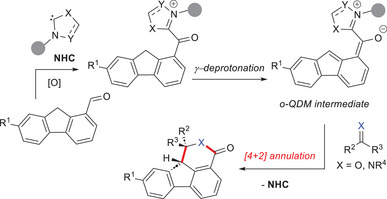
Strategy for the conversion of 9*H*‐fluorene‐1‐carbaldehydes into *o*‐QDMs for annulation reactions with ketones and imines.

An alternative approach has been recently devised by Biju and Qi groups, based on the usage of 9*H*‐fluorene‐1‐carbaldehydes under oxidative conditions.^[^ ^75^, ^76^ ^]^ The crucial acyl azolium suffers γ‐deprotonation generating the foreseen *o*‐QDM, which is then involved in [4 + 2] annulation with activated ketones and imines for the diastereo‐ and enantioselective synthesis of tetracyclic dihydro‐indenoisochromenones (tetracyclic δ‐lactones) and polycyclic dihydroisoquinolinones (Scheme 9). In those cases, the fluorenyl anion which is obtained after deprotonation has boosted aromatic character, which is responsible for the raised acidity of the benzylic proton of the starting aldehyde.

**Scheme 10:**
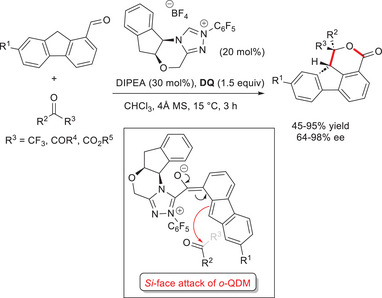
Enantioselective synthesis of tetracyclic δ‐lactones via *o*‐QDMs derived from 9*H*‐fluorene‐1‐carbaldehydes.

In Biju's work,^[^ ^75^ ^]^ chiral aminoindanol‐derived precatalyst with *N*‐pentafluorophenyl substituent (20 mol%) in combination with diisopropylethylamine (Hünig's base, DIPEA, 30 mol%) and **DQ** (1.5 equiv) was the best catalytic system for obtaining chiral tetracyclic δ‐lactones (53–87% yield, 64–98% ee, >20:1 dr) from an ample variety of (hetero)aryl trifluoromethyl ketones and 9*H*‐fluorene‐1‐carbaldehydes, comprised the aldehydes bearing 2‐naphthyl, 3‐furyl, 3‐thienyl, alkenyl, and alkynyl groups (Scheme 10). Likewise, 1,2‐diketones and α‐ketoesters have been used, with the expected products obtained in good yields (45–95%) and stereoselectivities (64–96% ee, up to > 20:1 dr). Scale‐up tests were undertaken for the reaction between 9*H*‐fluorene‐1‐carbaldehyde and 2,2,2‐trifluoroacetophenone (1.0 mmol), bringing the intended product with 90% ee (83% yield, >20:1 dr).

From the point of stereocontrol, it has been proposed that the ketone carbonyl group is attacked by the *Si*‐face of *o*‐QDM, which is undisturbed by the aminoindanol backbone of the catalyst. Besides, control experiments confirmed that the presence of the fluorenyl moiety is essential to form the *o*‐QDM intermediate, given that the reaction of 2,2,2‐trifluoroacetophenone with 2‐benzyl benzaldehyde produced no δ‐lactone product: it is plausible that the possible *o*‐QDM intermediate is hard to form due to the absence of stabilizing effects, which instead are assured by the fused five‐membered unit via the formation of a cyclopentadienyl moiety.

**Scheme 11:**
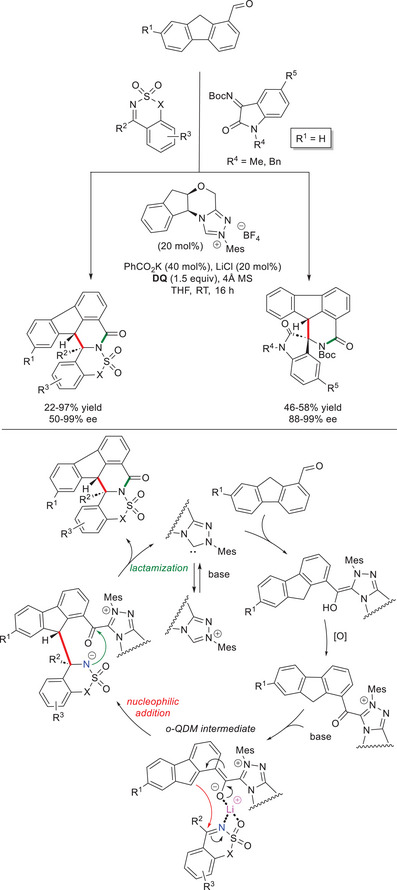
Synthesis of chiral polycyclic dihydroisoquinolinones via *o*‐QDMs derived from 9*H*‐fluorene‐1‐carbaldehydes.

Using *N*‐mesityl substituted chiral aminoindanol‐based triazolium salt (20 mol%), potassium benzoate (PhCO_2_K, 40 mol%), lithium chloride (LiCl, 20 mol%), and **DQ** (1.5 equiv), Qi and coworkers performed successful reactions of 9*H*‐fluorene‐1‐carbaldehydes with cyclic sulfonic imines, and isatin‐derived *N*‐Boc ketimines (Scheme 11).^[^ ^76^ ^]^ These experiments led the way to chiral polycyclic dihydroisoquinolinone scaffolds (22–97% yield, 50–99% ee) and spirocyclic compounds (46–58% yield, 88–99% ee) as single isomers, except for 1‐naphthyl substituted aldehyde (6:1 dr) and electron‐poor ketimine (5‐Br substituent, 2:1 dr). Also in this case, the practicality of the methodology was demonstrated by easy scalability of the model reaction between 9*H*‐fluorene‐1‐carbaldehyde (1.0 mmol) and ethyl benzo[*d*]isothiazole‐3‐carboxylate 1,1‐dioxide (1.5 mmol), with the polycyclic adduct afforded in 76% yield and 90% ee. Additionally, an useful post‐synthetic transformation (i.e., deprotection) has been achieved without eroding the optical purity of the starting material. In terms of mechanism, nucleophilic addition of NHC‐bound *o*‐QDM to the imine acceptor is likely driven by the coordination of lithium cation to *o*‐QDM, azomethine nitrogen, and one sulfonic oxygen atom. The intermediate that ensues gives rise to intramolecular lactamization releasing the NHC catalyst and the target compound.

**Scheme 12:**
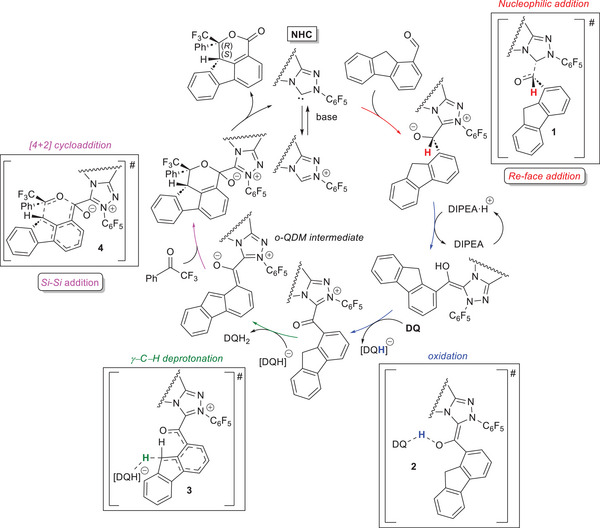
Calculated mechanistic pathway for the reaction of 2,2,2‐trifluoroacetophenone with 9*H*‐fluorene‐1‐carbaldehyde via *o*‐QDM intermediate.

Theoretical investigation on the reaction between 9*H*‐fluorene‐1‐carbaldehyde and 2,2,2‐trifluoroacetophenone was carried out by Wang and coworkers, in order to elucidate both mechanism and origin of stereoselectivity.^[^ ^77^ ^]^ Using a density functional theory (DFT) approach, it has been evidenced that the initial attack of the NHC catalyst occurs on the *Re*‐face of the aldehyde reagent (transition state **1**), then a sequence of protonation and deprotonation steps mediated by diisopropylethylammonium (DIPEA·H^+^)/diisopropylethylamine (DIPEA) couple leads to Breslow intermediate (Scheme 12). Subsequent oxidation goes through hydride transfer to **DQ** (transition state **2**), and the resulting acyl azolium intermediate is readily deprotonated at γ‐carbon to form *o*‐QDM species (transition state **3**). Subsequent [4 + 2] cycloaddition with the ketone partner gives rise to a zwitterionic intermediate (transition state **4**), from which NHC and the final lactone are released. The *RS*‐configuration of the latter has been shown to arise from the most favorable nucleophilic addition of the *Si*‐face of *o*‐QDM to the *Si*‐face of the ketone, with an expected enantioselectivity of 99.9%, in line with that observed experimentally (90% ee). Noncovalent index (NCI) and atoms‐in‐molecules (AIM) analyses helped understand that strongest weak interactions found in transition state **4**, namely lone pair (LP)^…^π, C─H^…^F, C─H^…^O, and π^…^π stacking, are most likely the chief factors determining the observed stereoselectivity.

**Scheme 13:**
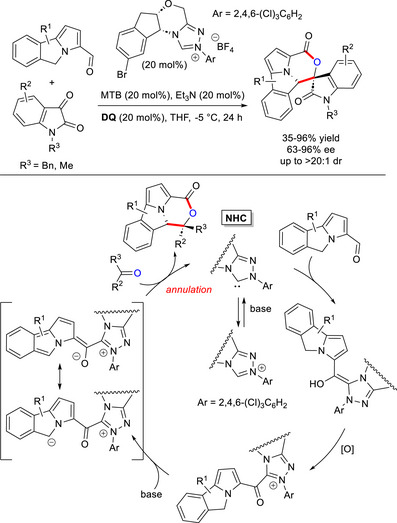
Synthesis of polycyclic morpholines via NHC‐catalyzed activation of remote C(sp^3^)─H of 5*H*‐benzo[*a*]pyrrolizine‐3‐carbaldehydes.

In 2023, the group of Ren and Chi disclosed the NHC‐catalyzed activation of a remote C(sp^3^)─H bond under oxidative conditions, deploying 5*H*‐benzo[*a*]pyrrolizine‐3‐carbaldehydes as proper substrates.^[^ ^78^ ^]^ In the present case, deprotonation of the NHC‐bound acyl azolium at C6 gives origin to a carbanion producing a key 12π species, that joins in the reaction with an *N*‐protected isatin (2π electrophile) to construct a polycyclic morpholine compound with a spirocyclic oxindole core (Scheme 13). Successful results in terms of yields (35–96%) and stereoselectivities (63–96% ee, up to > 20:1 dr) have been achieved using chiral aminoindanol‐derived precatalyst with 2,4,6‐trichlorophenyl substituent (20 mol%), magnesium di‐*tert*‐butoxide (MTB, 20 mol%), **DQ** (20 mol%), and Et_3_N (20 mol%) as additive (THF, ‐5 °C), with a broad scope of aldehydes and isatins found. The usefulness of this approach has been proven by large‐scale synthesis (1 mmol) of the adduct between 5*H*‐benzo[*a*]pyrrolizine‐3‐carbaldehyde and *N*‐benzyl isatin, without losing yield (86%) and stereoselectivities (82% ee, >20:1 dr), eventually followed by synthetic elaboration via saponification of the lactone unity (88% yield, 82% ee, >20:1 dr). It should be emphasized that the greater part of the target molecules exhibited encouraging antibacterial activities against the two plant pathogens *Xanthomonas axonopodis* pv. *citri* (*Xac*) and *Xanthomonas oryzae* pv. *oryzae* (*Xoo*),^[^ ^79^ ^]^ and some of them unveiled superior in vitro bioactivities compared to the commercial agrichemicals thiodiazole copper and bismerthiazol.

**Scheme 14:**
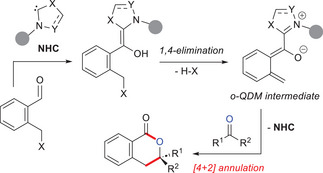
1,4‐Elimination route for the formation of NHC‐bound carbocyclic *o*‐QDMs for annulation reactions with ketones.

Glorius and Rovis groups exploited the use of a Breslow intermediate embedding a good leaving group so to obtain the *o*‐QDM species by 1,4‐elimination, thereby eluding the use of a γ‐acidic acyl azolium intermediate.^[^ ^80^, ^81^ ^]^ Once formed, *o*‐QDM is coupled with activated ketones to give 1‐isochromanone frameworks by [4 + 2] annulation (Scheme 14).

**Scheme 15:**
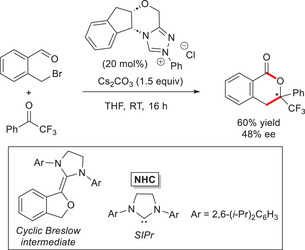
First example of NHC‐catalyzed enantioselective synthesis of 1‐isochromanones from 2‐(bromomethyl)benzaldehyde and fluorinated acetophenone.

In 2016, preliminary studies by Glorius and coworkers demonstrated that simple 2‐(bromomethyl)benzaldehyde reacted effectively with 2,2,2‐trifluoroacetophenone using *N*‐phenyl substituted chiral aminoindanol‐derived triazolium salt (20 mol%) and Cs_2_CO_3_ (1.5 equiv), yielding the target compound in 48% ee (Scheme 15).^[^ ^80^ ^]^ Basing on Berkessel's work on the isolation of Breslow intermediates, azolium enolates and NHC‐derived homoenolates,^[^ ^29^, ^30^ ^]^ attempts have been made to isolate the *o*‐QDM intermediate using a catalytically inactive NHC (1,3‐bis(2,6‐diisopropylphenyl)imidazolidin‐2‐ylidene, SIPr), but cyclic Breslow intermediate has always been obtained (100% conversion) as a result of intramolecular S_N_2 displacement.

**Scheme 16:**
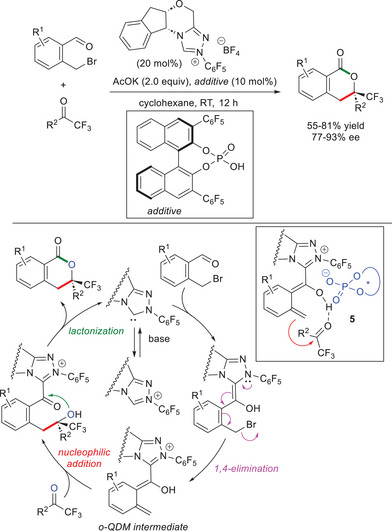
Synthesis of chiral 1‐isochromanones from 2‐(bromomethyl)benzaldehydes and trifluoromethyl ketones by NHC/Brønsted acid co‐catalysis.

Next, Rovis and Chen adopted NHC/Brønsted acid cooperative catalysis by use of *N*‐pentafluorophenyl substituted chiral triazolium pre‐catalyst (20 mol%), potassium acetate (AcOK, 2.0 equiv), and a chiral phosphoric acid (10 mol%), with the isochromanone products obtained in up to 93% ee (Scheme 16).^[^ ^81^, ^82^ ^]^

In the cooperative NHC/Brønsted acid catalyzed approach, the *o*‐QDM species is supposed to react with the carbonyl counterpart via an ion pair‐like transition state **5**, which implies that the conjugate phosphate anion may act as a chiral counterion: after intermolecular C─C bond formation, an intramolecular lactonization takes place, simultaneously liberating the NHC catalyst. However, other mechanistic pathways cannot be excluded.

### 2.2 Activation of Oxygen and Nitrogen Atoms via NHC‐Bound *o*‐QM, *aza*‐*o*‐QM, *aza*‐Fulvene, and Triaza‐Diene Intermediates

**Scheme 17:**
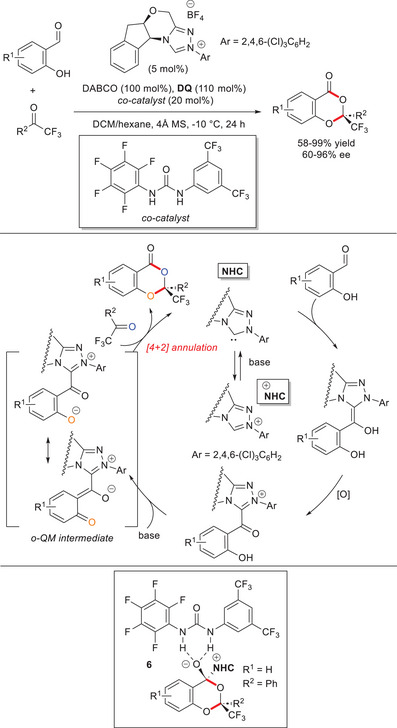
Use of NHC‐bound *o*‐QM intermediates for the synthesis of chiral ketal‐like adducts.

In 2017, an NHC‐bound *o*‐QM was designed by Chi and colleagues as the key species for remote activation (functionalization) of oxygen atom.^[^ ^83^ ^]^ Thus, 2‐hydroxy aryl aldehydes were converted into enantiomerically enriched ketal‐like compounds (60–96% ee) by reaction with both (hetero)aryl and alkyl trifluoromethyl ketones, using an aminoindanol‐derived chiral triazolium salt (5 mol%), 1,4‐diazabicyclo[2.2.2]octane (DABCO, 100 mol%), an achiral urea co‐catalyst (20 mol%), and **DQ** (110 mol%) (Scheme 17). This strategy was shown to be suitable for large‐scale synthesis (1.5 g) of the adduct resulting from the reaction between 2‐hydroxy‐5‐methylbenzaldehyde (5.1 mmol) and 2,2,2‐trifluoroacetophenone (5 mmol), with only 1 mol% catalyst sufficient to assure the same levels of performance (98% yield) and stereoselectivity (92% ee). Moreover, manganese dioxide (MnO_2_, 500 mol%) was reliably used as the terminal oxidant in combination with catalytic **DQ** (10 mol%),^[^ ^84^ ^]^ under identical reaction conditions.

As depicted in Scheme 17, a Breslow intermediate is initially formed from the aryl aldehyde and the NHC catalyst; then, oxidation to the corresponding acyl azolium and deprotonation of the latter on the phenol OH group lead to the pivotal *o*‐QM species, which participates in an *oxa*‐[4 + 2] annulation reaction with the ketone counterpart. In‐depth DFT studies on the model reaction between 2,2,2‐trifluoroacetophenone and salicylaldehyde demonstrated that the annulation step occurs in a concerted fashion, wherein ring formation and release of NHC take place simultaneously. An energetically most favored transition state **6** leading to the major (*S*)‐enantiomer was calculated in the presence of the urea co‐catalyst: the latter establishes hydrogen bond (H‐bond) interactions with the carbonyl oxygen atom of *o*‐QM and beneficial attractive π^…^π stacking between the pentafluorophenyl moiety and the indane framework of the catalyst. In addition, the urea group and the phenyl residue on the ketone give rise to relevant attractive dispersion interactions, which are presumably responsible for the obtained enantioselectivity.

It's notable that some promising compounds were obtained with antifungal activities against Eggplant *Verticillium*,^[^ ^85^ ^]^ *Phytophthora infestans*,^[^ ^86^ ^]^ and *Fusarium oxysporum*.^[^ ^87^ ^]^

**Scheme 18:**
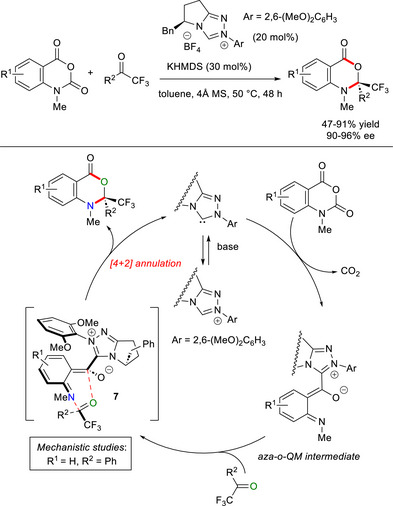
Enantioselective synthesis of dihydrobenzoxazin‐4‐ones via *aza*‐*o*‐QM intermediates derived from *N*‐methyl isatoic anhydrides.

Analog NHC‐bound *aza*‐*o*‐QM intermediates were obtained from *N*‐methyl isatoic anhydrides by NHC addition/decarboxylation, and used in *aza*‐[4 + 2] cyclization reactions with trifluoromethyl ketones to provide enantioenriched dihydrobenzoxazin‐4‐ones (90–96% ee, Scheme 18).^[^ ^88^ ^]^ The ideal catalytic system involved pyrrolidine‐based chiral triazolium salt with *N*‐(2,6‐dimethoxyphenyl) substituent (20 mol%) and potassium bis(trimethylsilyl)amide (KHMDS, 30 mol%) in toluene solvent, which has been applied to a wide range of ketones, including (hetero)aryl/alkyl ones and α‐ketoesters, while isatins did not react at all. DFT simulations were performed to gain insights into both mechanism and stereocontrol, showing that a [4 + 2] concerted process happens via the transition structure **7**, that displays favorable electrostatic interactions between the NHC‐bound *o*‐QM and the CF_3_ group in the ketone.

**Scheme 19:**
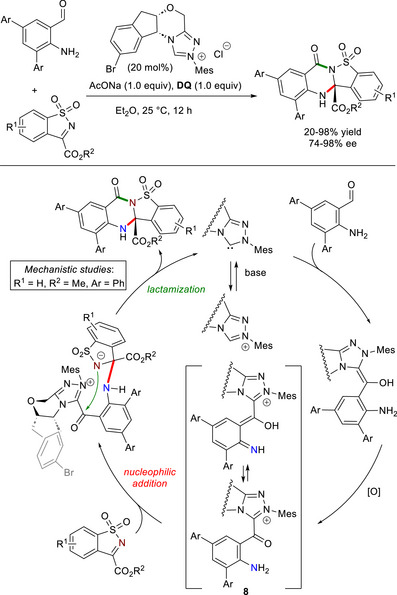
Synthesis of chiral quinazolinones through NHC‐catalyzed activation of “aniline‐like” N–H group.

Diaryl *o*‐aminobenzaldehydes were recently exploited by Wu and Zheng to activate an “aniline‐like” N–H group under NHC catalysis.^[^ ^89^ ^]^ In such a case, an acyl azolium is formed under oxidative conditions, then the *o*‐amino group reacts with cyclic sulfonic imines to form chiral quinazolinones (Scheme 19), having fluorescence properties useful for applications as chiral organic optical materials. Mechanistic studies led the authors to propose a possible path which involves nucleophilic attack of the activated *N*‐atom on the (favored) *Re‐*face of the electrophilic imine, meanwhile the *Re*‐face of the *N*‐nucleophile is locked by the chiral portion of the NHC catalyst. And on top of this, DFT calculations shed light on the much higher acidity of the N─H bond of intermediate **8** (pK_a_ = 15.69) with respect to the N─H moiety of the starting aldehyde (pK_a_ = 32.54). Poor to excellent yields (20–98%) and good to excellent enantioselectivities (74–98% ee) were achieved using the catalytic system formed by *N*‐mesityl substituted aminoindanol‐derived chiral triazolium salt (20 mol%), sodium acetate (AcONa, 1.0 equiv), and **DQ** (1.0 equiv), and wide substrate scope and scalability (1.0 mmol) have been also demonstrated.

**Scheme 20:**
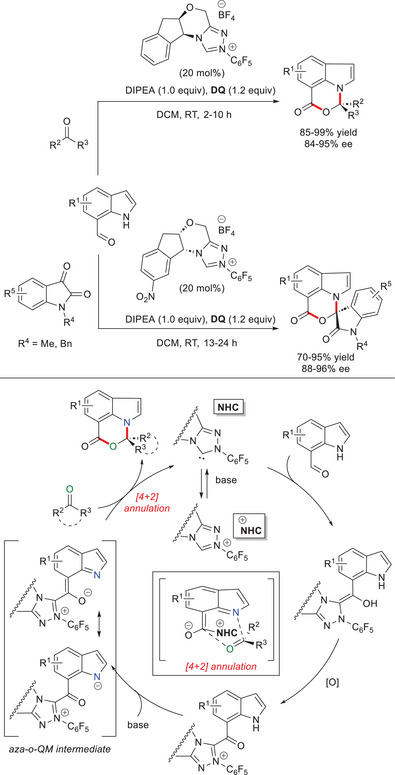
Synthesis of chiral *N*,*O*‐acetals via NHC‐bound *aza*‐*o*‐QM intermediates derived from indole‐7‐carboxaldehydes.

Activation of the indole N–H moiety was successfully achieved by dearomative deprotonation of the acyl azolium obtained by oxidation of the Breslow intermediate arising from addition of NHC to the remote formyl functionality of an indole‐7‐carboxaldehyde.^[^ ^90^ ^]^ Thereby, an analog *aza*‐*o*‐QM species was formed, and eventually involved in a formal [4 + 2] reaction with ketones to yield *N*,*O*‐acetal products bearing pyrroloquinazoline or oxazinoindole motifs (Scheme 20).^[^ ^91^ ^]^ More specifically, 3‐, 4‐, 5‐, and 6‐substituted indole‐7‐carboxaldehydes were treated with (hetero)aryl/alkyl trifluoromethyl ketones, difluoromethyl ketones, and α‐ketoesters, under the action of *N*‐pentafluorophenyl substituted aminoindanol‐derived chiral NHC precursor (20 mol%), Hünig's base (1.0 equiv) and **DQ** (1.2 equiv), giving excellent yields (85–99%) and enantioselectivities (84–95% ee) of the target scaffolds. In exactly the same conditions, it was also possible to apply imine reagents, and scale‐up synthesis has been addressed with a smallest catalyst loading (5 mol %) using 6‐chloro indole‐7‐carboxaldehyde (3.0 mmol) and 2,2,2‐trifluoroacetophenone (94% yield, 93% ee). Furthermore, use of *N*‐substituted isatin derivatives and an analog nitro‐substituted NHC catalyst opened the way to chiral spirocyclic *N*,*O*‐acetals in 70–95% yield, with 88–96% ee.

**Scheme 21:**
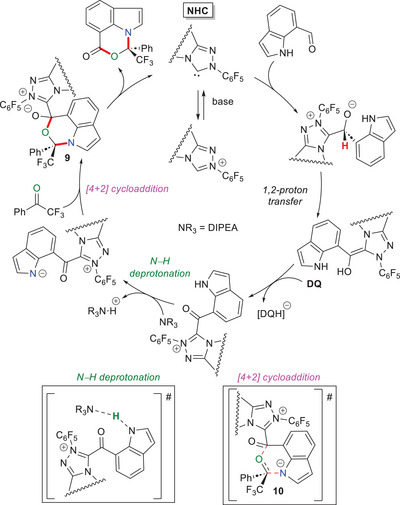
Calculated mechanistic pathway for the reaction of 2,2,2‐trifluoroacetophenone with indole‐7‐carboxaldehyde.

With computational and theoretical (DFT) calculations, Mondal and coworkers wrote more information on both the mechanism and origin of stereoselectivity in NHC‐catalyzed N–H activation/annulation reaction of indole‐7‐carboxaldehyde and 2,2,2‐trifluoroacetophenone.^[^ ^92^ ^]^ In analogy with the related NHC‐catalyzed remote C–H activation/annulation reaction of the same exact ketone with 9*H*‐fluorene‐1‐carbaldehyde (Scheme 12),^[^ ^77^ ^]^ three energetically feasible stages bring to the fundamental acyl azolium intermediate, namely chemoselective nucleophilic addition of NHC to the aldehyde carbonyl group (*Re*‐face attack), Brønsted acid (DIPEA·H^+^)/Brønsted base (DIPEA)‐promoted simultaneous proton transfers, and **DQ**‐assisted oxidation (Scheme 21). Thereafter, deprotonation of the acyl azolium species with DIPEA generates the *aza*‐*o*‐QM analog, which partakes in the [4 + 2]‐cycloaddition with the aromatic ketone. This last event defines the stereoselectivity of the entire process through a concerted, highly asynchronous way: the *N*‐nucleophile strikes the *Si*‐face of the ketone, after which an intramolecular *O*‐nucleophilic addition to the carbonyl group of the acyl azolium moiety comes, producing the six‐membered zwitterionic intermediate **9**; the latter eventually decomposes to unlock the (*R*)‐configured product (calculated ee > 99%) and restore the active NHC catalyst. Analyses by conceptual density functional theory (CDFT) and frontier molecular orbital (FMO) theory proved the central role of NHC in raising the nucleophilicity of *aza*‐*o*‐QM species, due to the uplift of its HOMO energy.

Later investigations brought Wang and Zhang to similar findings,^[^ ^93^ ^]^ aside from the postulated formation of the (*R*)‐product via addition of *aza*‐*o*‐QM from the *Re* face of the ketone. On the basis of NCI pictures and topological analysis of bond critical points (BCPs), the observed stereochemical outcome has been ascribed to weak forces which stabilize the major transition state **10**, that is to say LP^…^π interactions between the NHC triazole ring and the CF_3_ moiety on the ketone, as well as C–H^…^F, C–F^…^O and C–F^…^F interactions.

**Scheme 22:**
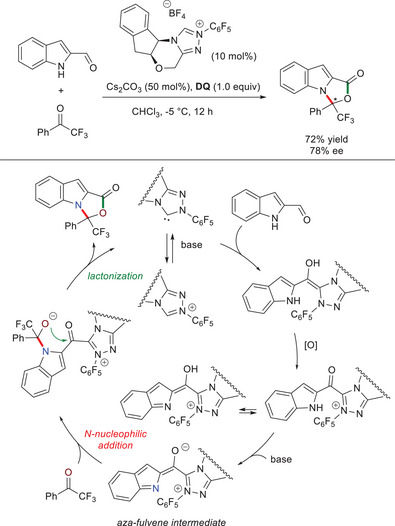
Annulation reaction of indole‐2‐carboxaldehyde with 2,2,2‐trifluoroacetophenone via *aza*‐fulvene type intermediate.

In 2020, studies by Biju, Jindal, and coworkers allowed to establish that cross‐conjugated *aza*‐trienolate (*aza*‐fulvene type) intermediates represent pivotal species to activate the nitrogen atom of indole‐ and/or pyrrole‐2‐carboxaldehydes toward annulation reactions with (hetero)aryl trifluoromethyl ketones, paving the way to *N*,*O*‐aminals.^[^ ^94^ ^]^ In this regard, a preliminary test in asymmetric series was performed by the reaction of indole‐2‐carboxaldehyde and 2,2,2‐trifluoroacetophenone with *N*‐pentafluorophenyl substituted chiral NHC pre‐catalyst (10 mol%), Cs_2_CO_3_ (50 mol%), and equimolar **DQ**, which resulted in the formation of an enantioenriched indole/oxazolone adduct (72% yield, 78% ee) (Scheme 22). Based on DFT computations, it has been assumed that the anticipated acyl azolium intermediate (or its enol tautomer) is deprotonated to generate the cross‐conjugated *aza*‐trienolate species; then, nucleophilic addition of the latter to the electrophilic ketone and following lactonization occur, with concomitant displacement of the NHC catalyst.

**Scheme 23:**
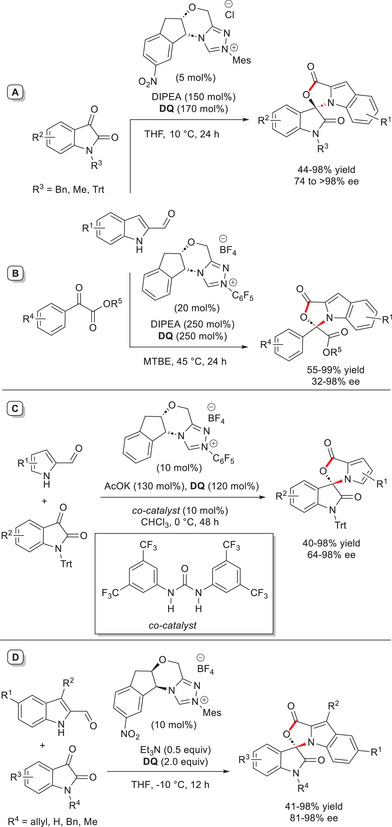
NHC‐catalyzed enantioselective annulation of indole‐ and pyrrole‐2‐carboxaldehydes with isatins and aryl α‐ketoesters.

Further research in the field of *N*‐activation by means of *aza*‐fulvene type intermediate was undertaken by other groups that same year. Jin and coworkers has rolled out an efficient strategy to transform indole‐2‐carboxaldehydes into enantioenriched *N*,*O*‐acetals by reaction with both *N*‐protected isatins and aryl α‐ketoesters.^[^ ^95^ ^]^ In the first instance, nitro‐substituted aminoindanol‐derived chiral triazolium salt with *N*‐mesityl substituent (5 mol%) was put in place jointly to Hünig's base (150 mol%) and **DQ** (170 mol%), with the target compounds obtained in 44–98% yield and up to > 98% ee using THF as the solvent (Scheme 23A). Otherwise, chiral *N*‐pentafluorophenyl substituted NHC pre‐catalyst (20 mol%) was the best choice for reacting α‐ketoesters, provided that the solvent was changed to *tert*‐butyl methyl ether (MTBE) and increased quantities of base and **DQ** (250 mol% each) were used. This adjusted catalytic system delivered the attended polycyclic products in 55–99% yield with 32–98% ee (Scheme 23B).

Concurrently, pyrrole‐2‐carboxaldehydes were tested as substrates for similar reactions with *N*‐trityl isatins, and best conditions were found using the catalytic system formed by aminoindanol‐derived chiral triazolium salt with *N*‐pentafluorophenyl group (10 mol%), AcOK (130 mol%), and **DQ** (120 mol%), in cooperation with 1,3‐bis[3,5‐bis(trifluoromethyl)phenyl]urea (10 mol%) (Scheme 23C). In the case in point, both position and type of substituent on the pyrrole‐2‐carbaldehyde skeleton had a strong influence on the results, with inactivation of the aromatic *N*‐nucleophilic center observed when an electron‐withdrawing group was located on C5.

Of importance are the antibacterial activities of the *N*,*O*‐acetal compounds against *Ralstonia solanacearum*,^[^ ^96^, ^97^ ^]^ responsible for blight with subsequent crops death: in some cases, the antibacterial effects have been shown to be superior over the two well‐established agrichemicals thiodiazole copper and bismerthiazol.

Hui and coworkers conducted very similar studies on the annulation reactions between indole 2‐carboxaldehydes and *N*‐protected isatins,^[^ ^98^ ^]^ slightly changing the experimental conditions: the triazolium salt enantiomer opposite to that used by Jin and colleagues ^[^ ^95^ ^]^ was exploited, with BF_4_¯ as counterion (10 mol%), along with Et_3_N (0.5 equiv) as the base and **DQ** (2.0 equiv) (THF, ‐10 °C), giving rise to (*S*)‐configured cyclic *N*,*O*‐aminal indoles in 41–98% yield and 81–98% ee (Scheme 23D).

It must also be mentioned that the group of Lan and Wang further studied the enantioselective heteroannulation of indole‐2‐carbaldehydes with alkyl, styryl, and (hetero)aryl fluorinated ketones, with best yields (40–90%) and enantioselectivities (69–94% ee) found when a thiourea additive was used alongside the NHC catalyst (not shown).^[^ ^99^ ^]^ Preferred formation of adducts with (*R*)‐configuration at the quaternary chiral center was theoretically predicted (DFT and NCI calculations) according to the lower energy of the parent transition state, due to significant stabilization of the deprotonated indolyl group arising from strong π^…^π attractive forces that involve the indolyl unit and the aryl moiety of the NHC catalyst. Such a conclusion was seemingly clarified by the higher reaction speed of electron‐poor aryl ketones or electron‐rich indoles, as inferred from kinetic experiments.

**Scheme 24:**
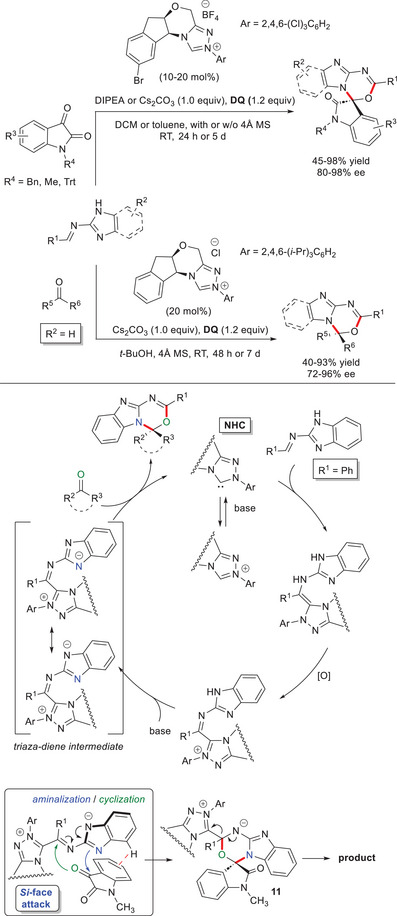
NHC‐catalyzed annulation reactions of (benz)imidazole‐derived aldimines with ketones via triaza‐diene intermediate.

Addition of chiral NHC to (benz)imidazole‐derived aldimines, followed by oxidation and proton transfer cleared the way for the attainment of triaza‐diene intermediates, useful for formal [4 + 2] annulation reactions with ketones (Scheme 24).^[^ ^100^ ^]^ An extensive library of chiral polyheterocyclic *N*,*O*‐acetals was attained under conditions very close to those reported for the reactions of NHC‐bound *aza*‐*o*‐QM^[^ ^90^ ^]^ and *aza*‐fulvene intermediates,^[^ ^94^, ^95^, ^98^ ^]^ using both acyclic ketones and isatins as the electrophilic counterparts of imine substrates derived from 2‐amino(benz)imidazoles. The final products were produced in highly satisfactory yields (up to 98%) and stereoselectivities (up to 98% ee), and relevant is that these results remained unaffected when **DQ** oxidant was replaced by inexpensive MnO_2_ (3.0 equiv), or synthesis was run on large scale (1.11 g of product) by only using 5 mol% catalyst.

On the back of experimental and theoretical (DFT) studies on the model reaction of 1‐methyl isatin with the imine derived from benzaldehyde and 2‐aminobenzimidazole, it was argued that an imidoyl azolium intermediate initially forms, as confirmed by HRMS (Scheme 24).^[^ ^101^ ^]^ Next, deprotonation of the remote N─H bond results in NHC‐bound triaza‐diene intermediate, that goes through an aminalization/cyclization sequence (concerted asynchronous addition) with the ketone reagent. The zwitterion **11** which forms delivers the NHC catalyst and frees up the annulation product. Perhaps, attack of the *N*‐nucleophile to the *Si*‐face of isatin directs the observed stereoselectivity, due to beneficial interaction (CH^…^π) that develops between the phenyl group of the imine and the aromatic ring of the isatin partner.

**Scheme 25:**
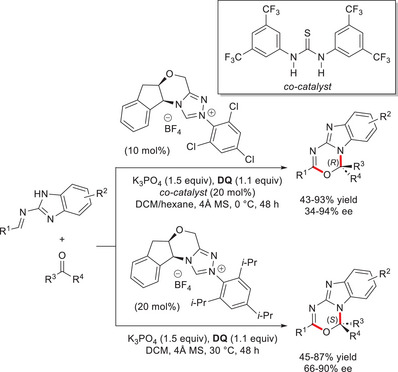
NHC‐catalyzed enantiodivergent synthesis of polyheterocyclic *N*,*O*‐acetals.

The strategy of using NHC‐bound triaza‐diene intermediates was further extended in the enantiodivergent synthesis of polyheterocyclic *N*,*O*‐acetals by the use of NHC catalysts having the same configuration, but different substituents at *N*‐atom.^[^ ^102^ ^]^ In details, chiral triazolium pre‐catalysts that bear *N*‐2,4,6‐triisopropylphenyl and *N*‐2,4,6‐trichlorophenyl residues were achieved from (1*S*,2*R*)‐1‐amino‐2‐indanol and used in the reaction of benzimidazole‐based aldimines with (hetero)aryl/alkyl trifluoromethyl ketones and phenyl ketones incorporating CF_2_H, CF_2_Cl and CF_2_Br motifs (Scheme 25). The catalytic system formed by the NHC precursor with *N*‐2,4,6‐triisopropylphenyl substituent (20 mol%), K_3_PO_4_ (1.5 equiv) and **DQ** (1.1 equiv) gave rise to (*S*)‐configured products (45–87% yield, 66–90% ee), while the opposite enantiomers (43–93% yield, 34–94% ee) arised from the use of *N*‐2,4,6‐trichlorophenyl substituted pre‐catalyst (10 mol%) along with 1,3‐bis[3,5‐bis(trifluoromethyl)phenyl]thiourea as a co‐catalyst (20 mol%), under little modified experimental conditions. It is noteworthy that this last protocol was also effective in reacting isatins (71–96% yield, 78–98% ee), however, not in an enantiodivergent fashion (not shown).

The key factors influencing the observed enantioselectivities were elucidated by mechanistic (DFT) investigation into the model reaction between 2,2,2‐trifluoroacetophenone and the imine made from 2‐aminobenzimidazole and benzaldehyde. Addition of the *N*‐nucleophile occurs preferentially to the *Si*‐face of the ketone carbonyl group, resulting in the formation of (*S*)‐stereoisomer via a transition state stabilized by C─H^…^F hydrogen bond interactions. Insertion of Cl atoms in the structure of the catalyst weakens these forces causing an enantioselectivity switch, and importantly all these results confirm that a fluorinated group is essential for achieving the enantiodivergence.

## 3 Summary and outlook

Foreground arising from long‐standing studies on NHC organocatalysis have spurred interest in the development of unconventional activation modes to open new routes for the stereoselective construction of C─C and C─heteroatom bonds. Within this framework, effective synthetic strategies have been achieved by the application of dearomative intermediates covalently bound to a chiral NHC catalyst, namely NHC‐tethered *o*‐QDMs, *o*‐QMs, and *aza*‐analogs (*aza*‐*o*‐QMs), as well as *aza*‐fulvene type and triaza‐diene intermediates. These species enabled successful (remote) activation of carbon, oxygen and nitrogen atoms incorporated in aromatic compounds toward annulation reactions with activated carbonyl and/or carbonyl‐like compounds, giving access to a wide variety of enantioenriched (poly)(hetero)cyclic molecules.

In most cases, these derivatives incorporate structural motifs that are widely common in biologically relevant compounds, such as natural products, pharmaceuticals, and agrochemicals. That's what can make this chemistry of especial importance in medicinal chemistry to drive the synthesis of potential drugs: one case on all is that of molecules containing trifluoromethyl and/or fluorurated fragments, as their incorporation could help to improve lipophilicity, permeability, and metabolic stability; and equally attractive are candidates embedding spirocyclic moieties, which may enhance both chemical‐physical and pharmacokinetic properties, as well as potency and selectivity. However, we must say that insight of the structure‐activity relationship (SAR) has not been dealt with in any of the discussed works.

Especially worth mentioning is the value of the reviewed methodologies for tackling the direct stereoselective synthesis of molecules having multiple stereocenters, including quaternary ones, which represents a still timely challenge for organic chemists, also in order to get well definite physical and biological properties.

But, on the other hand, despite the favorable results, some limitations exist, mainly in terms of starting substrates: just highly activated carbonyls are used, with the sole exception of imines to access triaza‐diene intermediates. And in addition to this, the use of a stoichiometric oxidant (Kharasch reagent) is required for the most part.

In face of the great potential that NHCs could offer in asymmetric reactions, we anticipate that the interest in using NHC‐bound dearomative intermediates for the stereoselective functionalization of (hetero)arenes would continue to grow, offering further opportunities for new substrates, activation modes, and target molecules. All this could be possibly done in conjunction with new developing concepts, including photocatalysis, electrochemistry, cooperative dual catalysis (e.g., with transition metals), and radical chemistry. And on top of that, new avenues might be offered by more sustainable approaches, perhaps taking advantage of renewable (bio‐based) starting materials, greener solvents and oxidants, or heterogeneous catalytic protocols for batch and flow reactions (process intensification). And, given the biological potential of the available compounds, one might also expect that future research will broaden the field of asymmetric NHC organocatalysis by including high‐throughput screening (HTS) and SAR studies of the obtained compound libraries, for lead generation and drug discovery.

## Conflict of Interest

The authors declare that they have no competing interests.
